# Supplementary material for: Nitric oxide orchestrates metabolic rewiring in M1 macrophages by targeting aconitase 2 and pyruvate dehydrogenase
Source: Nat Commun. 2020 Feb 4;11:698. doi: 10.1038/s41467-020-14433-7 (PMC7000728; doi:10.1038/s41467-020-14433-7)
Supplement: Supplementary file 1 — Supplementary figures and table with legends [file 41467_2020_14433_MOESM1_ESM.pdf]

Supplementary Information

Nitric Oxide Orchestrates Metabolic Rewiring in M1 Macrophages by Targeting Aconitase 2 and Pyruvate Dehydrogenase

Palmieri et al.

Supplementary Fig. 1

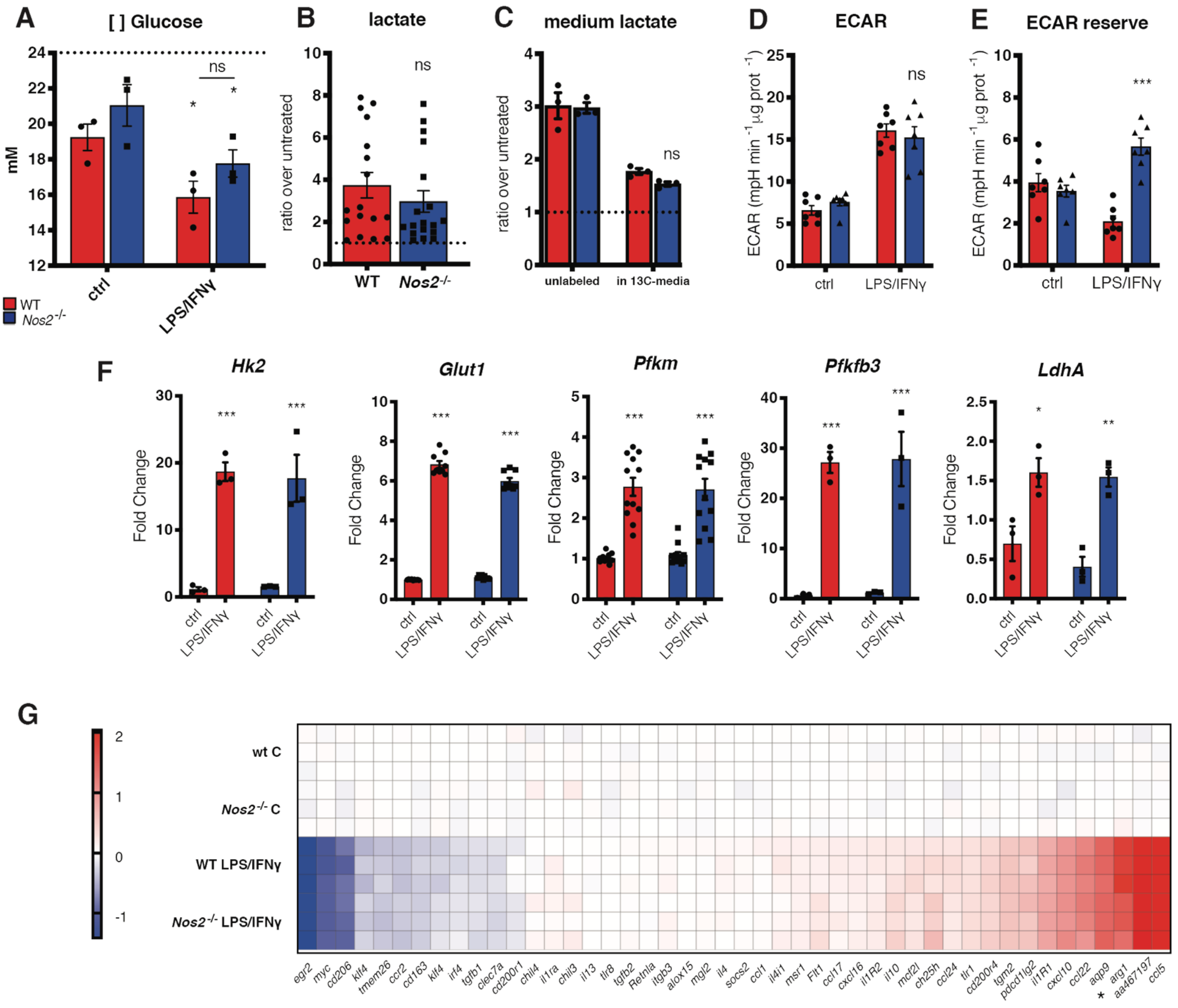

Supplementary Fig. 1. *Nos2*$^{-/-}$ macrophages show intact metabolism and inflammatory machinery. **A**) WT and *Nos2*$^{-/-}$ BMDMs were stimulated O.N. with LPS+IFNγ. Bar graphs show glucose concentrations measured in culture media by enzymatic reaction. Data (n=6) were analyzed by two-way ANOVA (Sidak's post-tests). Intracellular (**B**) and extracellular (**C**) lactate levels were quantified by GC-MS in cell extracts and in the media of O.N. stimulated WT and *Nos2*$^{-/-}$ BMDMs after culture for 4 additional hours with labeled tracer ([U-^{13}C] glucose). Data are normalized to the absolute peak area of the metabolite in WT control and were not significant by unpaired Student t-test (**B**) and two-way ANOVA (Sidak's post-tests) (**C**) (n=9). **D-E**) Bar graphs showing quantified, protein normalized, basal ECAR and ECAR reserve (difference between ECAR before and after oligomycin addition) from stress tests of O.N. stimulated WT and *Nos2*$^{-/-}$ macrophages. Data (n=9) were analyzed by two-way ANOVA with Sidak's post-tests and was significant for the treatments (p values<0.0001) but not for genotype in **D** but significant for both in **E** (p values= 0.0023, 0.0006, <0.0001). **F**) WT and *Nos2*$^{-/-}$ BMDMs were stimulated for 8h and mRNA was extracted from total cell lysates and analyzed by qPCR for *Hk2*, *Glut1*, *Pfkm*, *Pfkfb3* and *Ldha* expression. Data (n=6) were analyzed by two-way ANOVA with Sidak's post-tests (p

values=0.0004, 0.0005 (*Hk2*); <0.0001 (*Glut1*); <0.0001 (*Pfkm*); 0.004 (*Pfkfb3*); 0.0102, 0.0027 (*Ldha*)). **G**) Expression profile of selected anti-inflammatory genes from microarray analysis shown as heatmap of log10 fold changes of LPS+IFNγ-stimulated WT and *Nos2*$^{-/-}$ BMDMs compared to WT ctrl (n=3). All error bars display mean ± SEM. Source data are provided as a Source Data file.

Supplementary Fig. 2

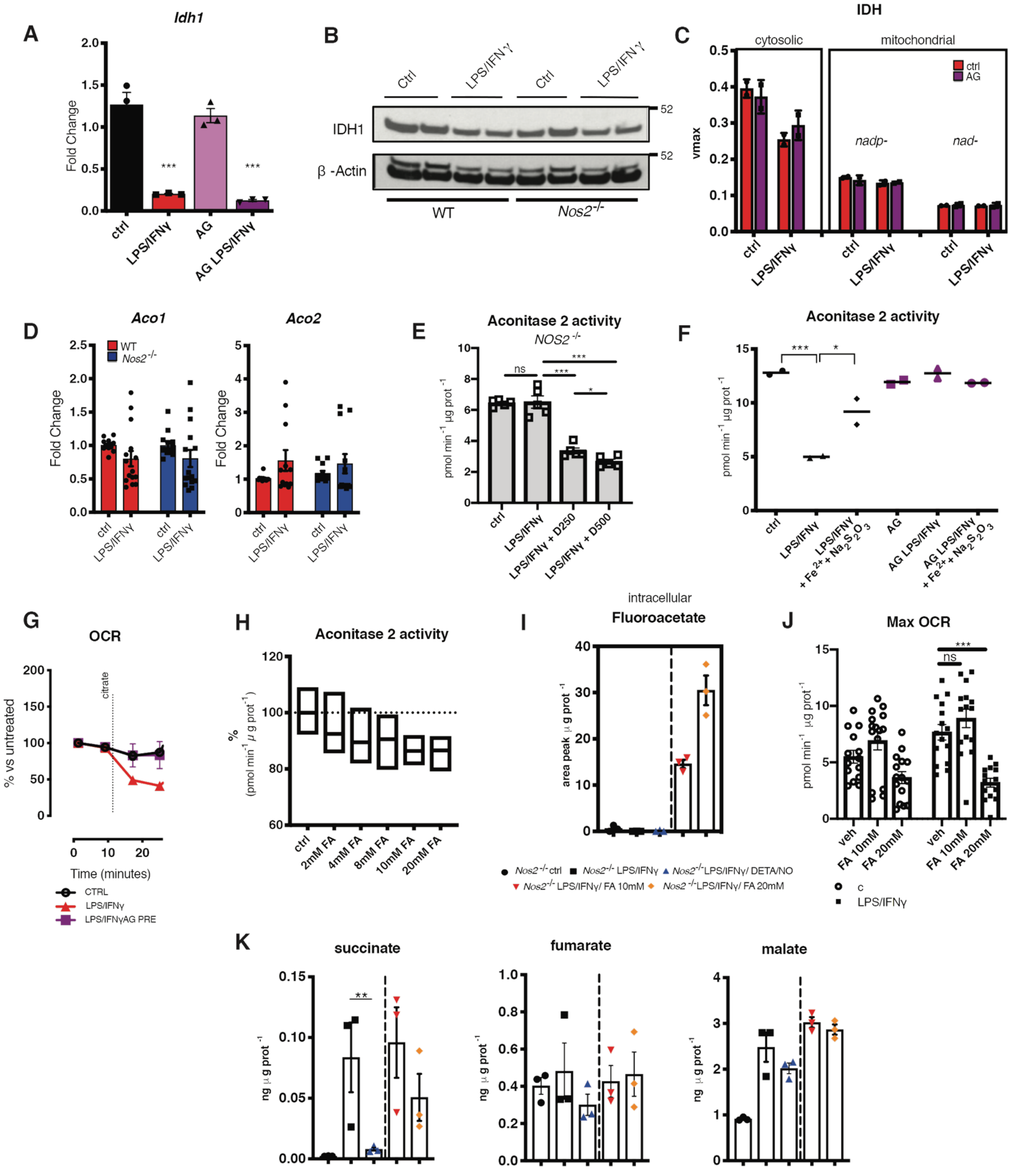

Supplementary Fig. 2. The TCA Break is due to NO targeting of mitochondrial Aconitase**.** **A**) WT BMDMs were stimulated O.N. in the presence of iNOS inhibitor aminoguanidine (AG), mRNA was extracted from total cell lysates and analyzed by qPCR for *Idh1* expression. Data (n=3) were analyzed by one-way ANOVA with Dunnett's multiple comparisons test (p value <0.0001). **B**) IDH1 immunoblots of whole macrophage extracts from 2 independent WT and *Nos2*$^{-/-}$, stimulated with LPS+IFNγ for 24h. β-actin was used as loading control (n=6). **C**) Cytosolic and mitochondrial IDH activities were assessed in O.N. stimulated BMDMs, pre-treated with AG (n=3). Vmax is shown. Data were not significant by one-way ANOVA (Tukey's post-tests). **D**) RNA as in **2A** were analyzed for *Aco1* and *Aco2* expression. Data (n=6) were not significant by two-way ANOVA (Sidak's post-tests).

E) Aconitase 2 activity from *Nos2*$^{-/-}$ stimulated with LPS+IFNγ in the presence of DETA/NO (250 and 500 μM). Data (n=6) were analyzed by one-way ANOVA with Dunnett's post-tests.

F) Aconitase 2 activity from WT and AG-pretreated BMDMs stimulated O.N. in the presence of ferrous iron ($FeSO_4$) and antioxidant (sodium thiosulfate, $Na_2S_2O_3$). Data were analyzed by one-way ANOVA (p =0.0003) with Tukey's post-tests (n=2). **G**) Line graph showing OCR from Seahorse analysis of O.N.-stimulated WT BMDMs or treated with AG after 9h of stimulation (24h total stimulation). Citrate, tartronate, ADP and PMP were co-injected. **H**) Aconitase 2 activity in resting WT BMDMs with increasing concentrations of Fluoroacetate (FA) (n=4). **I)** *Nos2*$^{-/-}$ BMDMs were stimulated with LPS +IFNγ in the presence of DETA/NO (500 μM) or FA. Residual FA was quantified by ESI-LC/MS-MS (m/z= 77>57 quantifier, 77>33 qualifier) and reported as normalized peak area. **J)** Protein normalized maximal OCR (FCCP rate minus non-mitochondrial respiration) from stress tests of *Nos2*$^{-/-}$ macrophages treated with FA 1 hr prior to stimulation. Data (n=3) were analyzed by two-way ANOVA with Sidak's post-tests. **K)** *Nos2*$^{-/-}$ BMDMs were stimulated as in **I**. Metabolites were quantified by ESI-LC/MS-MS and reported as normalized total ng. Data (n=6) were analyzed by one-way ANOVA with Dunnett's post-tests. All error bars display mean ± SEM. Source data are provided as a Source Data file.

Supplementary Fig. 3

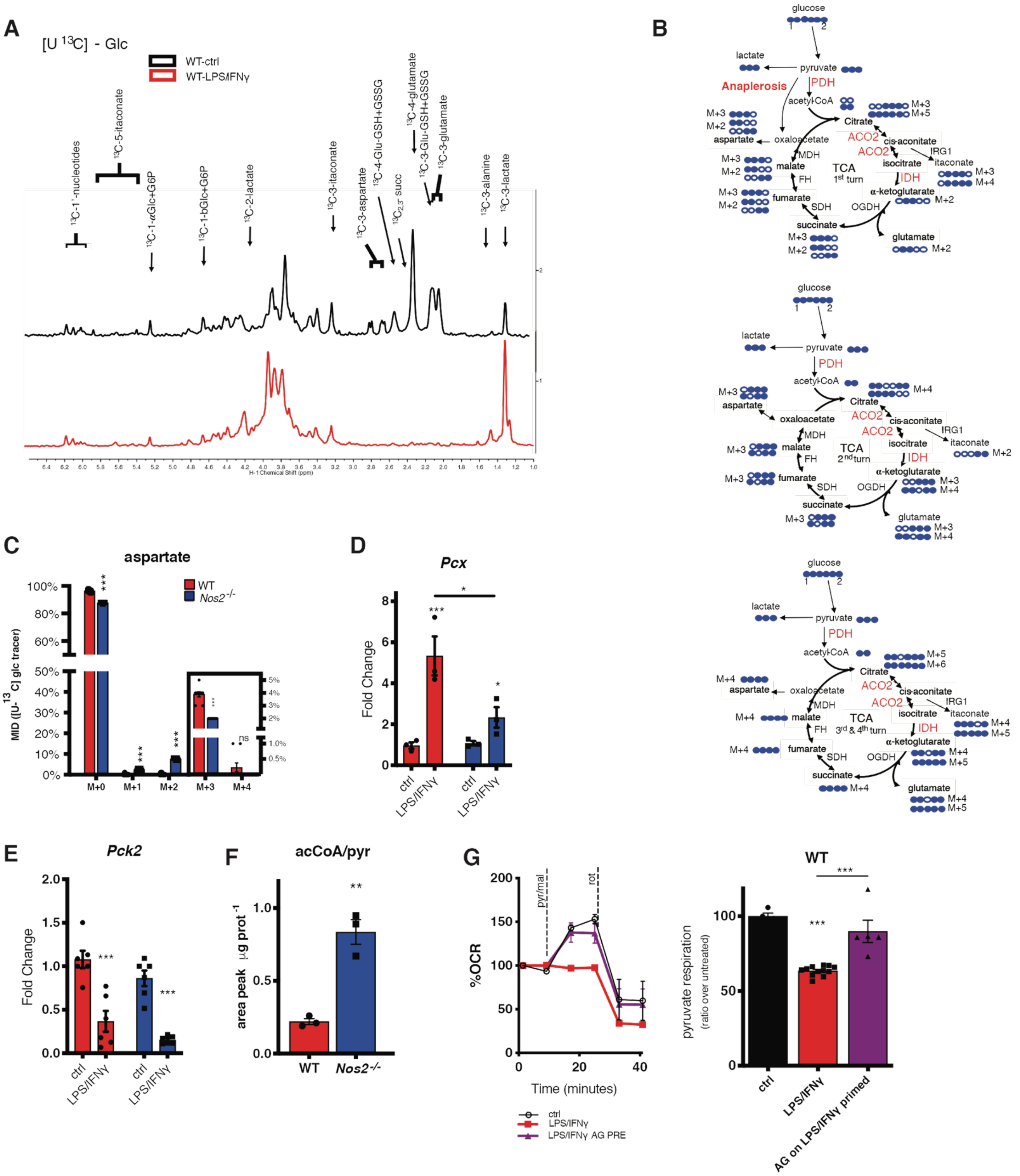

Supplementary Fig. 3. Absence of NO promotes pyruvate oxidation via PDH. **A**) Spectral comparisons of WT ctrl (black) versus WT LPS+IFNγ (red) are shown. The stacked 1-D HSQC spectra are representative of 2 experiments, n=3 biological replicate per group. **B**) Schematic illustration of qualitative changes in pathway contribution using [U-^{13}C] glucose as a tracer for determination of MIDs. **C**) MID of aspartate is shown. M+0 to M+6 indicate the different mass isotopologues as in 3B-I. Data (n=6) were analyzed by two-way ANOVA (interaction<0.0001) (Sidak's post-tests). (**D**-**E**) RNA as in 2A were analyzed for *Pcx* and *Pck2* expression. Data (n=6) were analyzed by two-way ANOVA with Sidak's post-tests (p values=0.0002, 0.0288, and 0.0143 between genotypes for Pcx, <0.0004 for Pck2). **F**) The acetyl-CoA to pyruvate ratio (acCoA/pyr) in WT and *Nos2*$^{-/-}$ BMDM after O.N. activation quantified by GC-MS. Data were significant by unpaired Student t-test (p=0.0021) (n=6). **G**) Line graph shows representative Seahorse analysis of permeabilized WT BMDMs stimulated for 24h or treated with AG after 9h from the stimulus (24h total stimulation) where state 3 OCR was elicited by pyruvate/malate to measure PDH flux. Bar graphs show quantified respiration as percentage of OCR relative to untreated cells. Data were analyzed by one-way ANOVA with Tukey's post-tests (p<0.0001) (n=3). All error bars display mean ± SEM. Source data are provided as a Source Data file.

Supplementary Fig. 4

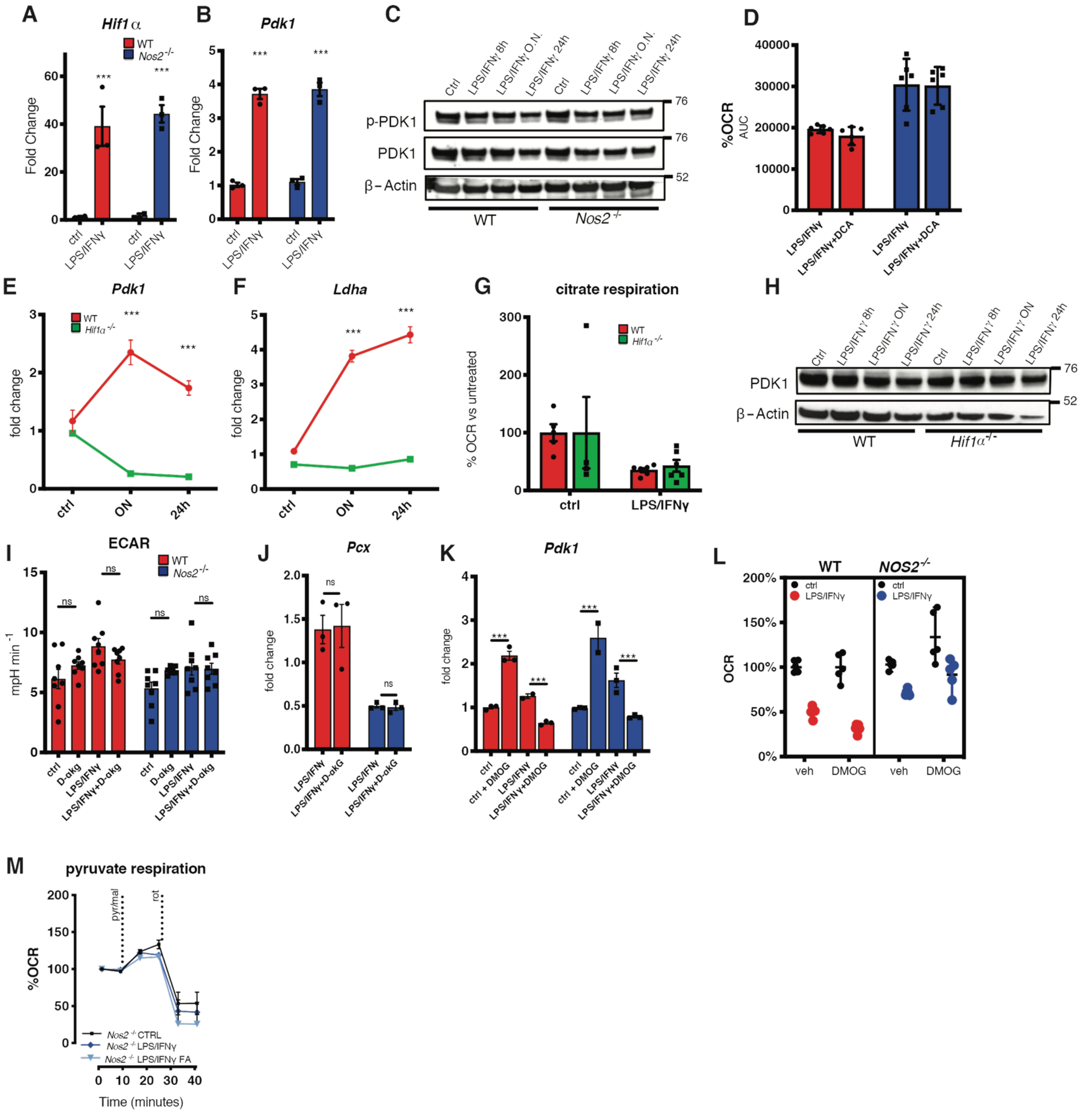

Supplementary Fig. 4. Decreased carbon flux through PDH is Hif1α independent. **A-B**) RNA as in 2A were analyzed for *Hif1α* and *Pdk1* expression. Data (n=6) were analyzed by two-way ANOVA with Sidak's post-tests (p values<0.0001 for Hif1α, 0.0063 for Pdk1). **C**) Immunoblots of phosphorylated and total PDK1 in whole macrophage extracts from WT and *Nos2*$^{-/-}$ stimulated at reported time points. β-actin was used as loading control (n=6). **D**) OCR in WT and *Nos2*$^{-/-}$ BMDMs

after addition of LPS+IFNγ. Bar graphs showing AUC (Area Under the Curve) after addition of dichloroacetate (DCA) after 8h of stimulation. Data were analyzed by two-way ANOVA with Sidak's post-tests (p values=0.0407, 0.09718). **E**-**F**) WT and *Hif1α*$^{-/-}$ BMDMs were stimulated with LPS+IFNγ; mRNA was extracted from total cell lysates at reported time points and analyzed by qPCR for *Pdk1* and *Ldha* expression. Data (n=3) were analyzed by two-way ANOVA (interaction<0.0001) (Sidak's post-tests). **G**) Quantification of exogenous citrate-dependent state 3 OCR in WT and *Hif1α*$^{-/-}$ BMDMs stimulated O.N. Data were analyzed by two-way ANOVA with Sidak's post-tests and were not significant between genotypes (n=2). **H**) PDK1 immunoblot in WT and *Hif1α*$^{-/-}$ BMDMs stimulated at reported time points. (n=6). **I**) Protein normalized basal ECAR from Seahorse stress tests of WT and *Nos2*$^{-/-}$ BMDMs pre-treated with dimethyl α-kG (D-αkG) 1 hr prior to O.N. stimulation. Data were not significant by two-way ANOVA (Tukey's multiple comparisons test) (n=3). **J**) WT and *Nos2*$^{-/-}$ BMDMs were pre-treated with D-αkG and stimulated for 8h. mRNA from total cell lysates were analyzed by qPCR for *Pcx*. Data were not significant by two-way ANOVA (Sidak's post-tests) (n=3). **K**) WT and *Nos2*$^{-/-}$ BMDMs were pre-treated with Dimethyloxalylglycine (DMOG) and stimulated for 8h. mRNA from total cell lysates was analyzed by qPCR for *Pdk1*. Data were analyzed by two-way ANOVA with Sidak's post-tests (p<0.0001) (n=3). **L**) Pyruvate/malate–respiration in presence of DMOG (n=4). Percentage increase of OCR upon fueling is shown relative to WT ctrl. **M**) Seahorse analysis of pyruvate/malate-OCR in *Nos2*$^{-/-}$ BMDMs after O.N. activation and pre-treatment with FA (n=3). All error bars display mean ± SEM. Source data are provided as a Source Data file.

Supplementary Fig. 5

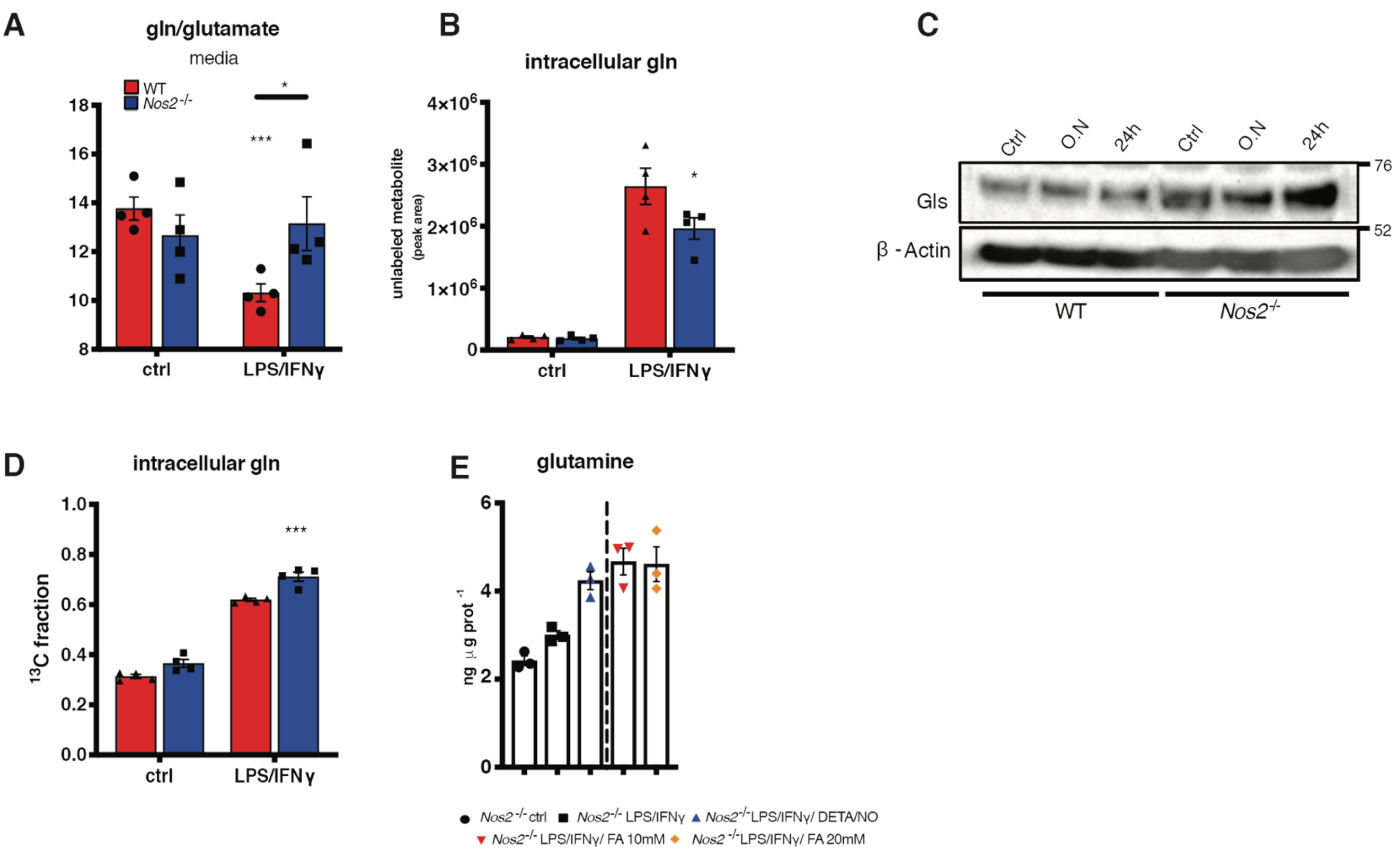

Supplementary Fig. 5. LPS/IFNγ increase glutamine utilization in NO dependent manner. Quantifications of intracellular glutamine levels by GC-MS in WT and *Nos2*$^{-/-}$ BMDMs stimulated O.N before (**A**) and after (**D**) culture for 4 additional hours with labeled tracer ([U-^{13}C] glutamine). Data were analyzed by two-way ANOVA with Sidak's post-tests (p values=0.0308, 0.0007) (n=4). **B**) Bar graphs show glutamine/glutamate ratios as indication of glutamine utilization, calculated from metabolite levels measured in culture media. Data (n=3) were analyzed by two-way ANOVA (interaction=0.0143) (Sidak's post-tests). **C**) Representative western blot for Glutaminase 1 (Gls) from whole cell lysates of WT and *Nos2*$^{-/-}$ BMDMs after activation with LPS+IFNγ for the indicated times. β-actin was used as loading control**. E)** *Nos2*$^{-/-}$ BMDMs were stimulated as in Fig. S2**I**. Glutamine was quantified by ESI-LC/MS-MS and is reported as normalized total ng. Data (n=6) were analyzed by one-way ANOVA with Dunnett's post-tests. All error bars display mean ± SEM. Source data are provided as a Source Data file.

Supplementary Fig. 6

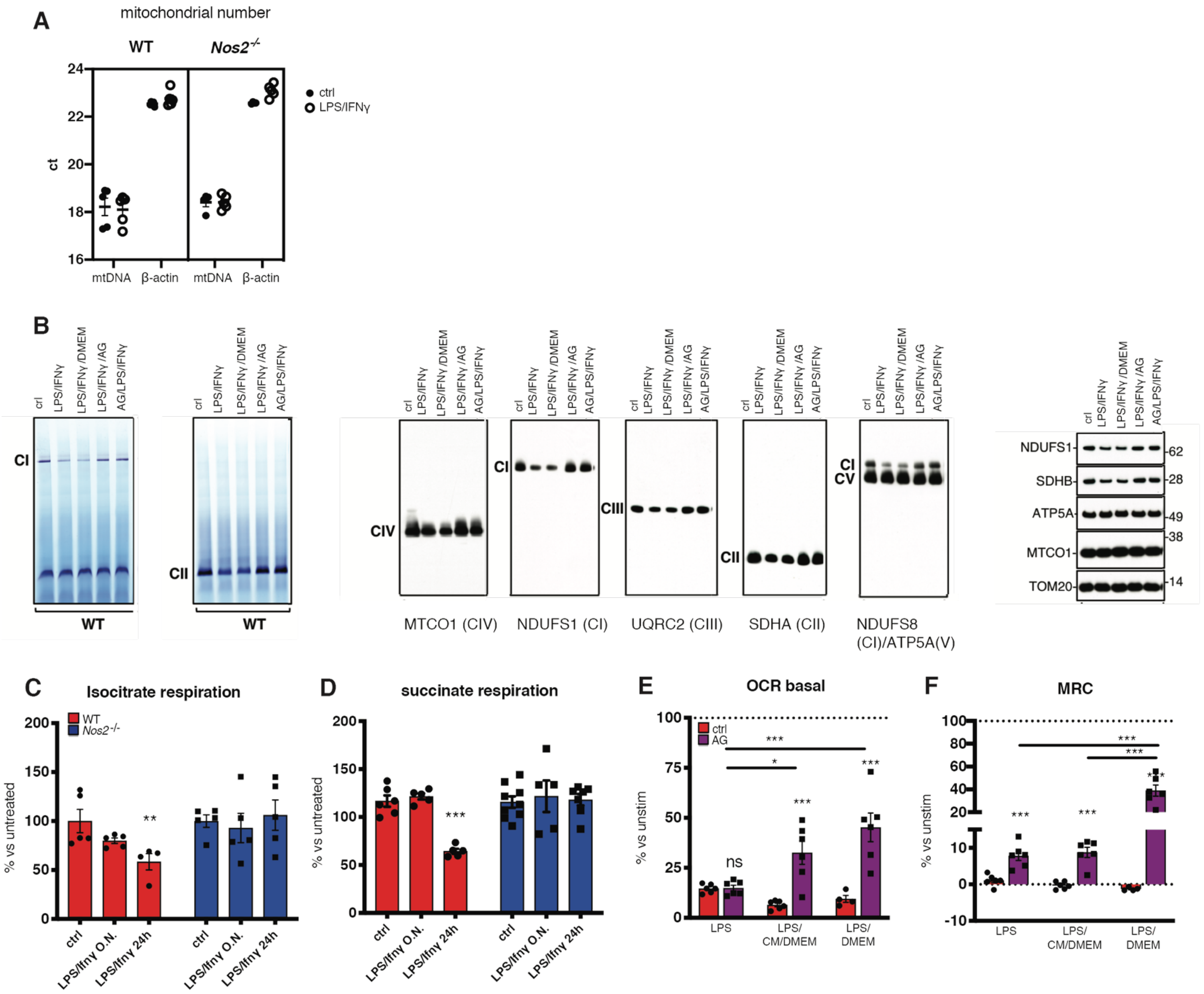

Supplementary Fig. 6. NO inhibits mitochondrial ETC complexes and promotes their loss. **B**) BMDMs from WT mice were stimulated for 24h (LPSIFNγ) or switched into fresh media and treated with AG (LPSIFNγ/AG) or vehicle (LPSIFNγ/DMEM) after 9h from the stimulus. AG/LPSIFNγ represents cells pre-treated with AG 1 hr prior to stimulation. CI and CII in-gel activity assays were performed together with native immunoblots to detect the CI subunit NDUFS1, the CII subunit SDHA, the CIII-Core protein 2 (UQCRC2), the CIV subunit I (MTCO1) and the CV alpha subunit (ATP5A) + NDUFB8 (CI). Subunits of ETC were also identified by SDS immunoblots were TOM20 was used as mitochondrial loading control. Quantification (percentage relative to ctrl cells) of exogenous isocitrate-dependent (**C**) or succinate-dependent (**D**) state 3 OCR in permeabilized WT and *Nos2*$^{-/-}$ BMDMs stimulated at reported time points measured with Seahorse Bioanalyzer. Data (n=6) were analyzed by two-way ANOVA with Tukey's (**C**) and Sidak's (**D**) post-tests. **E**) BMDMs from WT mice were seeded in Seahorse XF-96 cell culture microplates (0.8 x 10^5 cells/well). Cells were stimulated for 24h and/or treated with AG after 9h from the stimulus (24h total stimulation) with or without switching to partially conditioned media (CM) (half volume replaced with fresh media), or totally fresh media upon AG treatment (indicated as "LPS/CM/DMEM" and "LPS/DMEM" respectively); mitochondrial stress test was performed and basal OCR were plotted

and quantified in bar graphs. **F**) Maximal Respiratory Capacity (MRC) (maximal oxygen consumption rate attained adding the uncoupler FCCP) was quantified in the same conditions as **E)**. Data (**E-F**) are indicated as percentage relative to unstimulated cells. Data (n=4) were analyzed by two-way ANOVA (interaction<0.0001) (Sidak's post-tests). All error bars display mean ± SEM. Source data are provided as a Source Data file

Supplementary Fig. 7

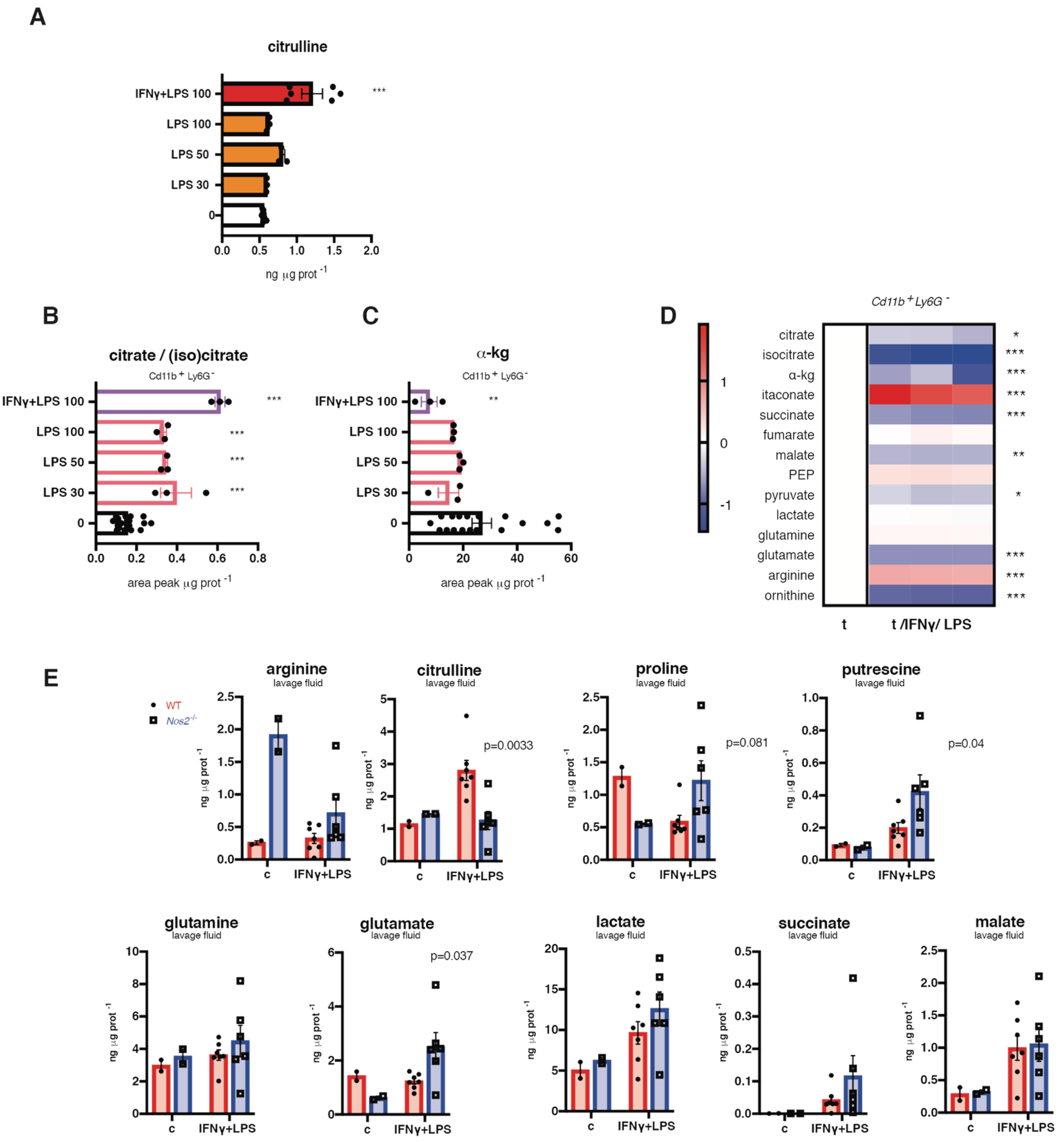

Supplementary Fig. 7. NO directs metabolic reprogramming *in vivo*. Mice were challenged as in Fig. 7A and peritoneal lavage was harvested and Cd11b+Ly6G- cells isolated, and both were subjected to metabolite extraction and ESI-LC/MS-

MS analysis. Normalized concentration of citrulline in the peritoneal lavage (**A)** and area peaks for selected intracellular metabolites (**B-C**) are shown. Data (n>3) were analyzed by one-way ANOVA with Dunnett's post-tests.
D) Heat-maps of the log10 ratio from the average peak areas from LC/MS-MS analysis of metabolites from Cd11b+Ly6G- cells isolated from WT mice challenged for Shwartzman reaction compared to thioglycolate alone as ctrl (t) (n=6). **E**) Absolute metabolite concentration from peritoneal lavage from mice under Shwartzman shock. ng/ tot µg prot are shown (n=8). Data (**E-F**) were analyzed by two way ANOVA with Sidak's post-tests. Shown p values indicate WT vs *Nos2*$^{-/-}$ comparisons in IFNγ+LPS condition. All error bars display mean ± SEM. Source data are provided as a Source Data file

Supplementary Table 1.

Canonical Pathway	z-score	p-value	Associated Gene Number
Phospholipase C Signaling	3.742	3.09E-04	15
B Cell Receptor Signaling	3.317	1.86E-03	11
Colorectal Cancer Metastasis Signaling	3.207	8.13E-04	14
NGF Signaling	3	7.94E-04	9
IL-8 Signaling	3	2.14E-02	9
Dendritic Cell Maturation	2.887	5.25E-04	12
cAMP-mediated signaling	2.887	3.55E-03	12
Paxillin Signaling	2.828	3.47E-04	9
Cholecystokinin/Gastrin-mediated Signaling	2.828	2.14E-03	8
Tec Kinase Signaling	2.828	2.63E-03	10
Ephrin Receptor Signaling	2.828	1.51E-02	9
Integrin Signaling	2.714	2.00E-03	12
ERK5 Signaling	2.646	4.57E-04	7
TREM1 Signaling	2.646	1.29E-03	7
Gαs Signaling	2.646	1.15E-02	7
HMGB1 Signaling	2.646	1.55E-02	7
Production of Nitric Oxide and Reactive Oxygen Species in Macrophages	2.53	7.41E-03	10
Role of Pattern Recognition Receptors in Recognition of Bacteria and Viruses	2.449	1.62E-03	9
FLT3 Signaling in Hematopoietic Progenitor Cells	2.449	5.37E-03	6
Melanocyte Development and Pigmentation Signaling	2.449	1.10E-02	6
p38 MAPK Signaling	2.449	1.38E-02	7
PAK Signaling	2.449	1.41E-02	6
Fcγ Receptor-mediated Phagocytosis in Macrophages and Monocytes	2.449	2.04E-02	6
HGF Signaling	2.449	3.02E-02	6
IL-6 Signaling	2.333	9.55E-04	9
GNRH Signaling	2.333	2.75E-03	9
ILK Signaling	2.309	8.13E-04	12
Oncostatin M Signaling	2.236	7.76E-04	5
Acute Myeloid Leukemia Signaling	2.236	8.71E-03	6
Wnt/Ca+ pathway	2.236	8.91E-03	5
Retinoic acid Mediated Apoptosis Signaling	2.236	1.26E-02	5
IL-17A Signaling in Airway Cells	2.236	1.41E-02	5
RANK Signaling in Osteoclasts	2.236	4.37E-02	5
FGF Signaling	2.236	4.37E-02	5
Death Receptor Signaling	2.121	8.71E-04	8
Lymphotoxin β Receptor Signaling	2	1.29E-03	6
Actin Nucleation by ARP-WASP Complex	2	7.24E-03	5
Neuregulin Signaling	2	1.55E-02	6
CNTF Signaling	2	2.57E-02	4
Thrombopoietin Signaling	2	3.89E-02	4
Activation of IRF by Cytosolic Pattern Recognition Receptors	1.89	3.72E-04	7
Prolactin Signaling	1.633	2.34E-04	8
Induction of Apoptosis by HIV1	1.633	1.86E-03	6
Type I Diabetes Mellitus Signaling	1.633	2.88E-03	8
VEGF Signaling	1.633	5.37E-03	7
Mouse Embryonic Stem Cell Pluripotency	1.633	1.74E-02	6
RhoA Signaling	1.414	5.62E-03	8
ERK/MAPK Signaling	1.414	2.34E-02	9
TNFR1 Signaling	1.342	6.31E-04	6
IL-9 Signaling	1.342	7.76E-04	5
CD40 Signaling	1.342	1.32E-02	5
Angiopoietin Signaling	1.342	1.58E-02	5
JAK/Stat Signaling	1.342	2.00E-02	5
Growth Hormone Signaling	1.342	2.09E-02	5
STAT3 Pathway	1.342	2.19E-02	5
BMP signaling pathway	1.342	2.57E-02	5
CD27 Signaling in Lymphocytes	1	2.57E-02	4
Role of NANOG in Mammalian Embryonic Stem Cell Pluripotency	1	3.39E-02	6
Wnt/β-catenin Signaling	0.816	1.20E-02	9
TWEAK Signaling	0.447	7.76E-04	5
Colorectal Cancer Metastasis Signaling	-1.087	2.75E-02	13
Pancreatic Adenocarcinoma Signaling	-1.008	1.38E-03	10
Chemokine Signaling	-0.943	5.50E-03	7
Cholecystokinin/Gastrin-mediated Signaling	-0.943	3.31E-02	7
LPS-stimulated MAPK Signaling	-0.873	2.29E-02	6
Melanocyte Development and Pigmentation Signaling	-0.873	4.17E-02	6
α-Adrenergic Signaling	-0.873	4.79E-02	6
RANK Signaling in Osteoclasts	-0.873	5.01E-02	6
Gαq Signaling	-0.831	3.47E-02	9
cAMP-mediated signaling	-0.823	3.47E-02	12
UVC-Induced MAPK Signaling	-0.797	8.32E-03	5
TNFR1 Signaling	-0.797	1.58E-02	5
Corticotropin Releasing Hormone Signaling	-0.797	1.91E-02	8
Inhibition of Angiogenesis by TSP1	-0.713	1.86E-02	4
Endothelin-1 Signaling	-0.676	3.63E-02	10
Induction of Apoptosis by HIV1	-0.673	2.14E-03	7
STAT3 Pathway	-0.582	2.29E-02	6
Ceramide Signaling	-0.582	3.39E-02	6
Production of Nitric Oxide and Reactive Oxygen Species in Macrophages	-0.537	2.09E-02	11
Glioma Signaling	-0.504	7.59E-04	10
Glioblastoma Multiforme Signaling	-0.504	3.31E-02	9
ATM Signaling	-0.478	3.24E-02	5
Hypoxia Signaling in the Cardiovascular System	-0.356	1.35E-02	6
PI3K/AKT Signaling	-0.291	3.31E-02	8
eNOS Signaling	-0.252	2.82E-02	9

Supplementary Table 1. Significantly enriched canonical pathways of differentially expressed genes. Ingenuity Pathway Analysis (IPA) was performed on data from Mouse 430 2.0 Array on 8h LPS+IFN**γ**-activated $Nos2^{-/-}$ vs WT BMDMs. The predicted functions are listed by decreasing z-score strength.
