# Supplementary material for: Nitric oxide orchestrates metabolic rewiring in M1 macrophages by targeting aconitase 2 and pyruvate dehydrogenase
Source: Nat Commun. 2020 Feb 4;11:698. doi: 10.1038/s41467-020-14433-7 (PMC7000728; doi:10.1038/s41467-020-14433-7)
Supplement: Supplementary file 3 — Reporting Summary [file 41467_2020_14433_MOESM3_ESM.pdf]

natureresearch

Corresponding author(s): Daniel McVicar

Last updated by author(s): Nov 22, 2019

Reporting Summary

Nature Research wishes to improve the reproducibility of the work that we publish. This form provides structure for consistency and transparency in reporting. For further information on Nature Research policies, see Authors & Referees and the Editorial Policy Checklist.

Statistics

For all statistical analyses, confirm that the following items are present in the figure legend, table legend, main text, or Methods section.

n/a	Confirmed	
☐	☒	The exact sample size (n) for each experimental group/condition, given as a discrete number and unit of measurement
☐	☒	A statement on whether measurements were taken from distinct samples or whether the same sample was measured repeatedly
☐	☒	The statistical test(s) used AND whether they are one- or two-sided<br>*Only common tests should be described solely by name; describe more complex techniques in the Methods section.*
☐	☒	A description of all covariates tested
☐	☒	A description of any assumptions or corrections, such as tests of normality and adjustment for multiple comparisons
☐	☒	A full description of the statistical parameters including central tendency (e.g. means) or other basic estimates (e.g. regression coefficient) AND variation (e.g. standard deviation) or associated estimates of uncertainty (e.g. confidence intervals)
☐	☒	For null hypothesis testing, the test statistic (e.g. F, t, r) with confidence intervals, effect sizes, degrees of freedom and P value noted<br>*Give P values as exact values whenever suitable.*
☒	☐	For Bayesian analysis, information on the choice of priors and Markov chain Monte Carlo settings
☒	☐	For hierarchical and complex designs, identification of the appropriate level for tests and full reporting of outcomes
☒	☐	Estimates of effect sizes (e.g. Cohen's d, Pearson's r), indicating how they were calculated

Our web collection on statistics for biologists contains articles on many of the points above.

Software and code

Policy information about availability of computer code

Data collection	Affymetrix 430 2.0 was used to collect microarray data.<br>Applied Biosystems 7300 was used to collect quantitative RT-PCR data.<br>XF-96 analyzer (Seahorse Bioscience) was used to perform energetics studies.<br>18.8 T spectrometer was used to aquire 1-D 1H{13C} HSQC NMR spectra of cell extracts, which were quantified using MNova software (Mestrelab Research).<br>A 7890A GC system (Agilent Technologies) combined with a 5975C Inert MS system (Agilent Technologies) were used to measure Isotopomer distributions and metabolite levels.<br>Agilent 6410B (Agilent Technologies) interfaced with a 1200 Series HPLC quaternary pump (Agilent Technologies) with MassHunter Quantitative B.07.01.sp was used to acquire and quantify targeted mass spectrometry data.
Data analysis	GraphPad Prism 7 was used to perform statistical analysis and plot results. Extracellular acidification rate (ECAR) and oxygen consumption rate (OCR) were analysed by Wave (Agilent Technologies, Inc.)<br>Partek Genomic Suite software was used to analyze microarray data and perform statistical analysis. IPA (Qiagen) was used for analysis of differential expressed genes.

For manuscripts utilizing custom algorithms or software that are central to the research but not yet described in published literature, software must be made available to editors/reviewers. We strongly encourage code deposition in a community repository (e.g. GitHub). See the Nature Research guidelines for submitting code & software for further information.

Data

Policy information about availability of data

All manuscripts must include a data availability statement. This statement should provide the following information, where applicable:

- Accession codes, unique identifiers, or web links for publicly available datasets
- A list of figures that have associated raw data
- A description of any restrictions on data availability

Microarray data has been deposited under the accession number GSE115120 and is available starting December 10, 2019. Metabolomics data is available at the NIH Common Fund's National Metabolomics Data Repository (NMDR) website, the Metabolomics Workbench, https://www.metabolomicsworkbench.org where it has been assigned Project ID PR000867. The data can be accessed directly via it's Project DOI: http://dx.doi.org/10.21228/M8MM6Z

Field-specific reporting

Please select the one below that is the best fit for your research. If you are not sure, read the appropriate sections before making your selection.

☒ Life sciences ☐ Behavioural & social sciences ☐ Ecological, evolutionary & environmental sciences

For a reference copy of the document with all sections, see nature.com/documents/nr-reporting-summary-flat.pdf

Life sciences study design

All studies must disclose on these points even when the disclosure is negative.

Sample size	3-9 mice per group were used for animal studies. No prior sample size calculation was performed. Group sizes for animal studied were established considering previous experience with yields of cell numbers and biological matrices.
Data exclusions	No data exclusions were made.
Replication	Results of experiments are based on representative results of at least 3 independent bone marrow derived macrophage cultures (for in vitro experiments)/cohorts of mice (for in vivo experiments) following the same protocol. All findings were replicated successfully.
Randomization	Animals were randomized by age for bone marrow extractions. For in vivo studies, mice were also randomized by bodyweight and sex prior to challenge with LPS.
Blinding	No blinding was performed.

Reporting for specific materials, systems and methods

We require information from authors about some types of materials, experimental systems and methods used in many studies. Here, indicate whether each material, system or method listed is relevant to your study. If you are not sure if a list item applies to your research, read the appropriate section before selecting a response.

Materials & experimental systems

n/a	Involved in the study	
☐	☒	Antibodies
☒	☐	Eukaryotic cell lines
☒	☐	Palaeontology
☐	☒	Animals and other organisms
☒	☐	Human research participants
☒	☐	Clinical data

Methods

n/a	Involved in the study	
☒	☐	ChIP-seq
☐	☒	Flow cytometry
☒	☐	MRI-based neuroimaging

Antibodies

Antibodies used	anti-Mouse antibodies for western blot:<br>from Abcam: Rabbit monoclonal anti-ACO2 (RRID: AB_11144142), Rabbit monoclonal anti-ACO1 (RRID: AB_11130595), Rabbit monoclonal anti-IDH1 (AB 172964), Rabbit polyclonal anti-Lamin B1 (RRID: AB_443298), Rabbit monoclonal anti-phospho-PDH E1 (AB177461), Anti-total OXPHOS Rodent (AB110413), Rabbit monoclonal anti-NDUFS1 (AB169540), Mouse monoclonal anti-SDHA (RRID: AB_301433), Mouse monoclonal anti-SDHB (RRID: AB_301432), Mouse monoclonal anti-MTCO1 (RRID: AB_2084810), Mouse monoclonal anti-ATP5A1 (RRID: AB_301447), Mouse monoclonal anti-UQCRC2 (RRID: AB_2213640), Rabbit monoclonal anti-NDUFB8 (AB192878), Anti-S-nitrosocysteine (RRID:AB_10697568), Anti-Glutaminase (RRID:AB_10561964).<br>from Millipore: Mouse monoclonal anti-Actin (RRID: AB_2223041)<br>from Sigma: Rabbit polyclonal anti-CS (RRID: AB_10604321)<br>from Proteintech: Rabbit polyclonal anti-FECH (RRID: AB_2231579)

from Novus Biologicals: Rabbit polyclonal anti-HIF1a (RRID: AB_10001045), Rabbit monoclonal anti-PDK1 (pSer241) (NBP1-96065)
from Santa Cruz Biotechnology: Mouse monoclonal anti-TOM20 (RRID: AB_2207538)
also used for immunoprecipitation: Recombinant Anti-Lipoamide Dehydrogenase (Abcam)(RRID:AB_2732908),

Validation

Antibody validation was according to manufacturer's website

Animals and other organisms

Policy information about studies involving animals; ARRIVE guidelines recommended for reporting animal research

Laboratory animals

C57BL/6J and NOS2 deficient mice (B6.129P2-Nos2tm1Lau/J purchased from the Jackson Laboratory) were maintained and bred in the Frederick National Laboratory Core Breeding Facility, aged 8-10 weeks at start of experiments.

Wild animals

Wild animals were not used

Field-collected samples

Field collected animals were not used

Ethics oversight

Mice were used in accordance with an approved protocol by the NCI Frederick Institutional Animal Care and Use Committee

Note that full information on the approval of the study protocol must also be provided in the manuscript.

Flow Cytometry

Plots

Confirm that:

- [ ] The axis labels state the marker and fluorochrome used (e.g. CD4-FITC).
- [ ] The axis scales are clearly visible. Include numbers along axes only for bottom left plot of group (a 'group' is an analysis of identical markers).
- [ ] All plots are contour plots with outliers or pseudocolor plots.
- [ ] A numerical value for number of cells or percentage (with statistics) is provided.

Methodology

Sample preparation

50 ul of cell culture media were used to measure cytokine levels with cytometric bead-based immunoassays (BD Biosciences) for IL-10 (558300), IL-6 (558301), TNFa (558299), MCP1 (558342), Mip1a (558449), IL-1b (560232), IL-12p40 (560151), KC (558340)

Instrument

BD LSRFortessa X-20 (Becton Dickinson)

Software

BD FACS Diva was used to used to acquire data and FCAP Array v3.0 software was used for analysis.

Cell population abundance

Describe the abundance of the relevant cell populations within post-sort fractions, providing details on the purity of the samples and how it was determined.

Gating strategy

According to manufacturer instructions

- [ ] Tick this box to confirm that a figure exemplifying the gating strategy is provided in the Supplementary Information.
